# The oncogenic gamma herpesviruses Epstein-Barr virus (EBV) and Kaposi's sarcoma-associated herpesvirus (KSHV) hijack retinoic acid-inducible gene I (RIG-I) facilitating both viral and tumour immune evasion

**DOI:** 10.1016/j.tvr.2022.200246

**Published:** 2022-08-20

**Authors:** Alana Nash, Elizabeth J. Ryan

**Affiliations:** a Department of Biological Sciences, Faculty of Science and Engineering, University of Limerick, Ireland; b Limerick Digital Cancer Research Centre, University of Limerick, Ireland; c Health Research Institute, University of Limerick, Limerick, V94 T9PX, Ireland

**Keywords:** Pathogen recognition, EBV, KSHV, Innate immune evasion, MDA5, RIG-I, Innate immunity, EBV, Epstein Barr virus, KSHV, Kaposi's sarcoma virus, RIG-I, retinoic-acid-inducible protein I, RLR, RIG-I-like receptors, MDA-5, melanoma differentiation-associated protein 5, ADAR1, adenosine deaminase acting on RNA 1, EBERs, Epstein-Barr virus (EBV)-encoded RNAs

## Abstract

Herpesviruses evade host immunity to establish persistent lifelong infection with dormant latent and replicative lytic phases. Epstein-Barr virus (EBV) and Kaposi's Sarcoma-associated virus (KSHV) are double-stranded DNA herpesviruses that encode components to activate RNA sensors, (Retinoic acid-inducible gene I (RIG-I) and melanoma differentiation-associated protein 5 (MDA5). Yet both viruses can effectively evade the antiviral immune response. The ability of these viruses to disarm RIG-I to evade immunity allowing viral persistency can contribute to the creation of a protected niche that facilitates tumour growth and immune evasion. Alternatively, viral nucleic acids present in the cytosol during the replicative phase of the viral lifecycle can activate pro-inflammatory signaling downstream of RIG-I augmenting tumour promoting inflammation.

Understanding how these viral proteins disrupt innate immune pathways could help identify mechanisms to boost immunity, clearing viral infection and enhancing the efficacy of immunotherapy for virally induced cancers. Here we review literature on the strategies EBV and KSHV use to either enhance or inhibit RLR signaling.

## 1 Introduction

Viruses hijack cellular function, interfering with host cell enzyme activity and regulatory complexes creating favourable conditions for their replication and spread. EBV and KSHV are oncogenic γ−herpesviruses capable of establishing life-long infection in the host. Both herpesviruses have distinct latent and lytic replication phases and alternating between these states coupled with phases of abortive lytic replication allows the establishment of a lifelong persistent infection and a complex relationship with the host [[1], [2], [3]]. EBV infects an estimated >90% of the adult population and is linked to diseases such as mononucleosis and multiple sclerosis [4]. The International Agency for Research on Cancer classifies EBV as a carcinogenic agent in seven cancers: nasopharyngeal cancer (NPC), stomach, Hodgkin lymphoma (HL), Non-Hodgkin lymphomas (NHL), Burkitt's lymphoma (BL), and lymphoepithelioma-like carcinoma (LELC). KSHV is a carcinogenic agent in three cancers; Kaposi's Sarcoma which is a cancer of the endothelium, and the B cell proliferative diseases primary effusion lymphoma (PEL) and the multicentric Castleman disease (MCD). Since the discovery of EBV and KSHV in 1964 and 1994, respectively, their viral genomes have been studied to find mechanisms of host cellular disruption that could promote malignancy [5].

RIG-I-like receptor (RLR) dependent signaling is important in primary viral infection, lytic reactivation, and tumour progression [6]. There are three RLR receptors, RIG-I, melanoma differentiation-associated protein 5 (MDA5) and Laboratory of Genetics and Physiology 2 (LGP2) [7]. RIG-I is a cytosolic dsRNA receptor, and when activated transmits a signaling pathway leading to the expression of type I interferons (IFN) and inflammatory cytokines. RIG-I is composed of a C-terminal DExD/H-box RNA helicase domain which binds to dsRNA, and a N-terminal caspase recruitment domain (CARD) which activates mitochondrial antiviral signaling protein (MAVs). MAVS recruits and allows activation of TANK-binding kinase 1 (TBK1) and inhibitor of nuclear factor kB kinase (IKK) which in turn phosphorylate signaling molecules and transcription factors, including NF- κB, interferon regulatory factor 3 (IRF3) and IRF7 [7,8]. Translocation of phosphorylated IRF3, IRF7 and NF-κB to the nucleus from the cytosol induce type I interferon (IFN) expression and other immunostimulatory genes [7,8]. Chronic inflammation arising from dysregulation of the NF-κB signaling pathway is associated with approximately 20% of cancers [9].

In this review, we will discuss the interactions of oncogenic γ−herpesviruses with RIG-I. EBV and KSHV have methods of either promoting or preventing RIG-I activation, dependent on viral life stage that could contribute to viral persistence and herpesvirus associated cancer progression.

## 2 Oncogenic γ−herpesviruses control activation of the RNA sensor RIG-I

**Fig. 1:**
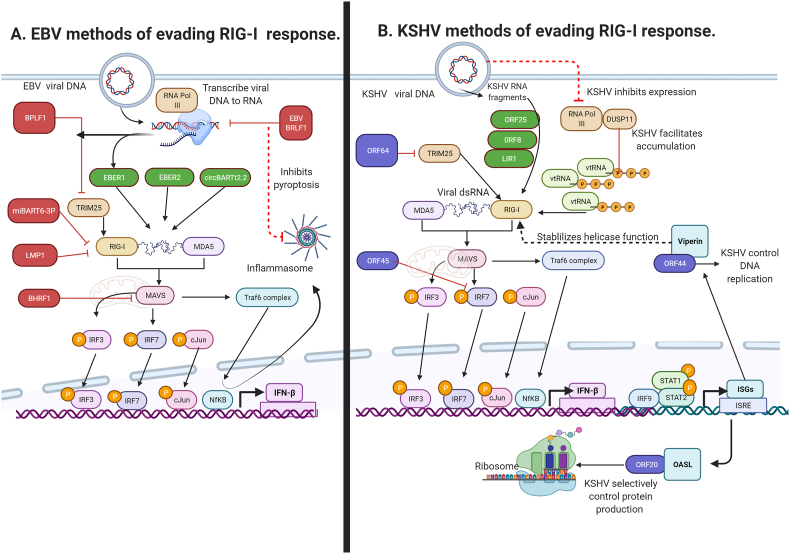
Mechanisms EBV and KSHV use to disrupt RIG-I dependent signaling. (a) RNA Pol III transcribes EBV DNA to RIG-I-activating RNAs, shown in green: EBER1, EBER2 and circBART2.2. EBV inhibitors of the RIG-I response are shown in red: miBART6-3p, LMP1, BRLF1, and BPLF1. EBV BPLF1 and KSHV ORF64 are homologs which disrupt RIG-I regulator TRIM25. (b) KSHV genomic fragments that activate RIG-I are shown in green: ORF25, ORF8, LIR1. KSHV activates RIG-I through accumulation of host 5'ppp-vtRNAs. KSHV proteins that interact with and alter function of RIG-I response proteins are shown in purple: ORF20, ORF45, ORF44 and ORF50. Created with BioRender.com.. (For interpretation of the references to color in this figure legend, the reader is referred to the Web version of this article.)

EBV and KSHV have different methods of achieving the same result: activating RIG-I (Fig. 1). RIG-I is activated by binding to viral or host short dsRNA with a 5'triposphatase (5'ppp) moiety in the cytosol [22]. In a manner conserved among herpesviruses, EBV utilizes host RNA Polymerase III (RNA Pol III) to transcribe viral DNA into RNA [23]. RNA Pol III transcribes the most abundant of latent EBV RIG-I activators: EBV-encoded small noncoding RNAs (EBERs) [23]. EBERs bind and activate RIG-I yet EBV proteins can degrade host-derived RNAs to prevent RIG-I activation. EBV encodes for a highly conserved viral host-shut off protein (vhs) that degrades host RNA5SP141 to prevent RIG-I activation in NPC [11]. EBV has methods for both promoting and preventing RIG-I activation (Fig. 1a).

KSHV lytic reactivation is restricted by RIG-I and MDA5 [24]. While RNA Pol III transcribes EBV DNA into RIG-I activating RNA, interestingly, KSHV activates RIG-I in an RNA Pol III-independent manner [12,23]. During KSHV infection, RIG-I is activated in by binding to KSHV genomic regions that form RNA fragments or to host derived RNAs [12,13]. Three KSHV fragments are particularly enriched for RIG-I binding; ORF25(43561–43650), ORF8(10420–10496) and the repeat region LIR1(119059–119204) [12]. These fragments are expressed during the lytic cycle, and each have high sequence dissimilarity which suggests that the specific KSHV dsRNA structure rather than the specific sequence motif is important for RIG-I binding [12].

Although the KSHV fragments are not directly transcribed by RNA Pol III, Zhao et al., suggest RNA Pol III plays a role in the innate immune response by transcribing host RNAs for pathogen recognition receptor (PRR) sensing in KSHV infection [13]. KSHV lytic infection facilitates cellular accumulation of host RIG-I-activating RNAs [13] Zhao et al., searched for RLR activating ligands in KSHV infection and found both host and viral RNAs, 650 and 1324 RNAs bound to RIG-I and MDA5, respectively. Host cellular vault RNAs (vtRNAs) were among the most enriched RNAs bound to RIG-I [13]. In BC-3 cells with lytic KSHV infection, vtRNAs with an immunostimulatory 5'ppp moiety (5'ppp-vtRNA) accumulate and activate RIG-I. 5′-ppp vtRNAs are capable of restricting KSHV lytic reactivation and inducing expression of IFNs and ISGs. In uninfected cells, host dual specificity phosphatase 11 (DUSP11) removes immuno-stimulatory 5'ppp moieties from vtRNAs. KSHV lytic reactivation reduces expression of DUSP11 and RNA Pol III facilitating cellular accumulation of RIG-I activating 5'ppp-vtRNAs [13].

However, KSHV has a reported method to prevent host-derived RNAs from activating the RLR pathway [25]. Adenosine deaminase acting on RNA 1 (ADAR1) plays a role in preventing aberrant activation of PRRs by host-derived RNAs in murine cell lines and mutations in ADAR1 are associated with diseases characterized with over production of IFN [26,27]. Zhang et al., reported ADAR1 is a pro-viral factor and is required for KSHV lytic reactivation. ADAR1 is suggested to reduce immunostimulatory RNAs, inhibiting RLR-dependent induction of IFNs [25]. ADAR1 does not edit vtRNAs and the unedited cellular RNAs, GINS1 and NOP14, are suggested as possible ADAR1 targets. Interestingly, both Zhao et al. and Zhang et al. found MDA5 to be a more important restricting factor to KSHV lytic reactivation than RIG-I [13,25].

Murine γHV68 (MHV68) which is a homolog of EBV and KSHV also manipulates RIG-I signaling. He et al., reported a method conserved by MHV68, HSV-1 and KSHV to activate RIG-I via deamidation [18]. Herpesviral vGAT is a homolog of glutamine amidotransferase (GAT), a deamidase important for biosynthesis of an assortment of metabolites. vGAT and cellular phosphoribosyl-formylglycinamide synthase (PFAS) share homology, yet the viral homolog lacks enzymatic activity. It is suggested that vGAT ‘activates’ PFAS, recruits PFAS to RIG-I, deamidates and activates RIG-I. This was the first report of PRR activated by an enzyme [18]. MHV68 can use the activated RLR pathway to further viral persistence. MHV68 is reported to manipulate the downstream transcription factor IKKβ to promote phosphorylation of the viral transcription factor RTA (replication transactivator) and promote phosphorylation of RelA to degrade NF-kB, preventing antiviral cytokine expression [[19], [20], [21]]. These studies on MHV68 suggest that hijacking the RIG-I response is an important survival strategy for γherpesviruses.

To summarise, RIG-I and MDA5 restricts KSHV lytic reactivation. However, when the KSHV undergoes lytic replication and the immunostimulatory KSHV genomic fragments are expressed, cellular DUSP11 and ADAR1 are co-opted to reduce the level of RIG-I activation by host derived RNAs. As described in Table 1, while EBV and KSHV have different methods of promoting and preventing RIG-I activation to allow viral immune escape. Both oncogenic *y-*herpesviruses exert a tight level of control on RIG-I activation (Fig. 1, Table 1).

**Table 1:** EBV and KSHV genes that interact with host RIG-I response proteins leading to viral immune escape and oncogenic mechanisms.

	Function in Viral Lifecycle	Immune Escape and Oncogenic Mechanisms	References
EBV			
EBERs (EBV-encoded small non-coding RNAs)	• Most abundant latent proteins expressed	• Apoptotic resistance	^8,19,22,30^
	• Activate RIG-I and TLR3	• Inflammation	
	• Suppress PKR	• Angiogenesis	
	• Induce expression of IL-32-mediated pro-inflammatory cytokines, TNF-α, IL-1, and IL-6, and cell-type specific growth factors (IGF-1, IL-9, IL-10)	• Proliferation	
		• Immunosuppressive tumour microenvironment (TME) by excluding T and NK cells	
**CircBART2.2**	• Bind and activates RIG-I	• Immunosuppressive TME: T cells inhibited	^12^
	• Induce expression of pro-inflammatory cytokines	• Inflammation	
	• Regulates PD-L1 expression in NPC		
**MiBART6-3p**	• Binds and degrades RIG-I mRNA	• Facilitates viral replication and immune escape	^15^
**LMP1** (Latent Membrane Protein 1)	• Promotes degradation of RIG-I on the proteasome pathway	• Immunosuppressive TME, disrupted memory T cell expansion and differentiation	^36,37^
	• Constitutively activates NF-κB	• Inflammation	
	• Suppresses IRF3 phosphorylation, IFN-β and ISRE promoter activation		
**BHRF1**	• Targets mitochondria	• Apoptotic resistance	^39^
	• Inhibits MAVs		
**BRLF1**	• Interacts with RNA Pol III	• Immunosuppressive TME	^14^
	• Prevent RIG-I inflammasome activation	• Selective control of protein production	
**EBV BPLF1/KSHV ORF64**	• Inhibits TRIM25	• Facilitates viral replication and immune escape	^13,41,42^
	• Inhibits RIG-I activation		
***KSHV***			
**ORF44**	• Stabilise RIG-I helicase activity	• Promote viral DNA replication	^45^
	• Hijack ISG product Viperin to a pro-viral factor		
**ORF20**	• RIG-I activation upregulates OASL	• Promotes viral replication through selective control of protein production	^44^
	• Hijacks ISG product OASL to a pro-viral factor		
**ORF45**	• Inhibits IRF7 phosphorylation	• Facilitates viral replication and immune escape	^43^
	• Inhibits type I IFN production		

Toll-like receptor 3 (TLR3), Protein kinase R (PKR), Interleukin-32 (IL-32), Tumour necrosis factor-alpha (TNF-a), Interleukin-6 (IL-6), Natural Killer (NK) cells, nuclear factor kappa beta (NF-κB), Interferon regulatory factor 3 (IRF3), Interferon stimulated response elements (ISRE), programmed death ligand 1 (PD-L1), nasopharyngeal cancer (NPC), Tripartite Motif 25 (TRIM25), B cell lymphoma 2 gene (BC-L2), oligoadenylate synthase-like protein (OASL), Open reading frame (ORF), Interferon stimulated gene (ISG).

## 3 EBV encodes RIG-I activators

### 3.1 EBV-encoded RNAs (EBERs) activate RIG-I signaling

EBERs were the first reported DNA virus-encoded RNAs to bind and activate RIG-I [10]. EBERs are highly conserved among EBV isolates and are the most abundantly expressed viral transcripts in EBV latency, about 10^7 copies per cell [10,[28], [29], [30]]. EBERs are non-polyadenylated viral noncoding RNAs that are 167 nt (EBER1) and 172 nt (EBER2) in length. While EBER1 and EBER2 share only 54% primary sequence similarity, they form similar dsRNA-like molecules with a stem-loop structure by intermolecular base-pairing [31]. This dsRNA-like structure allows for interaction and activation of PRRs Toll-like receptor 3 (TLR3) and RIG-I [10,32]. It is interesting that EBV encodes for two conserved ds-RNA 5'ppp-like moieties that activate RNA PRRs. EBERs participate in EBV manipulation of host innate immune responses, they induce expression of pro-inflammatory chemokines and cytokines, and enhance apoptotic resistance [21,25]. EBER1 and EBER2 are potentially cell type specific and can induce autocrine growth factor expression e.g., insulin-like growth factor 1 (IGF-1), Interleukin (IL)-9 in T-lymphocytes and IL-10 in B-lymphocytes [[33], [34], [35], [36], [37]]. EBERs can activate the RIG-I pathway in monocyte-derived macrophages, inducing expression of IL-6, TNF-α and immunosuppressive indoleamine 2,3-dioxygenase (IDO) [38]. The presence of EBERs in BL cells induce resistance to IFN-α-mediated apoptosis by binding and inhibiting phosphorylation of protein kinase R (PKR) [39,40].

EBER-induced activation of the RIG-I dependent inflammatory response is critical to tumour progression in NPC [28]. RIG-I and EBER2 expression were positively corelated in NPC *in vitro* and *in vivo* [28} Interestingly, in this study RIG-I dependent activation of tumour associated macrophages promoted xenograft growth. A further study using an NPC cell line, demonstrated EBERs expressed in extracellular vesicles activated RIG-I/TLR3 pathways in neighbouring epithelial cells and promoted angiogenesis through VCAM-1 expression [41]. EBER-induced activation of RIG-I signaling aids a tumour permissive microenvironment, tumour growth, and tumour progression (Table 1/Fig. 1).

### 3.2 EBV circBART2.2 can bind to RIG-I and add to a tumour permissive environment through exclusion of T cells

Bam-HI A rightward transcripts (BARTs) are highly expressed during latent EBV infection and are found in all EBV-positive cells and associated tumours [42,43]. BARTs include long non-coding RNAs (lncRNAs), viral microRNAs (miRNAs) and circular RNAs (circRNAs). CircRNAs are a class of non-coding RNAs with a covalent closed-loop structure lacking 5′ or 3’ ends. CircBARTs regulate gene expression by functioning as competitive endogenous RNAs [42]. CircBARTs expression is often dysregulated in different cancers [44,45]. BARTs play a role in regulating expression of EBV latency-associated genes to avoid immune detection in immunocompetent individuals and facilitate tumour immune escape in immunocompromised individuals [14,17,42]. EBV circBART2.2 binds RIG-I and modulates the immune response leading to chronic inflammation, a tumour permissive microenvironment, and viral replication [14,17].EBV circBART2.2 binds to the helicase domain of RIG-I and activates transcription factors NF-κB and IRF3 [14]. These activated transcription factors can induce oncogenic inflammation and upregulate programmed death ligand 1 (PD-L1) expression in NPC [14]. PD-L1 is expressed on the surface of immunosuppressive cells in the tumour microenvironment and often on tumour cells. PD-L1 interacts with PD-1 on T cells, acting as an immune checkpoint to prevent T cells with specific tumour antigens from activating and killing tumour cells [14]. EBV acts through RIG-I signaling to aid a tumour permissive environment by induction of the inflammation response and the exclusion of T cells.

## 4 EBV encodes RIG-I inhibitors

### 4.1 EBV miRNA, BART6-3p, represses RIG-I response

BARTs are known to modulate immune response for viral proliferation by regulating EBV and host gene expression. EBV encodes for a RIG-I inhibitor, BART6-3p [17]. EBV BART6-3p is reported to degrade the 3′UTR of RIG-I mRNA, allowing viral immune escape. Micro RNAs (miRNAs), including BART6-3P, are highly expressed in NPC during EBV latency [17]. EBV miBART6-3p represses host expression of RIG-I response genes including IFN-*B* and resulting phosphorylation of TBK1 and STAT1 [17].

### 4.2 EBV latent membrane protein 1 (LMP1) degrades RIG-I via the proteasome pathway

The EBV genome has two oncogenic proteins, Latent Membrane Protein 1 (LMP1) and BRLF1, that are reported to inhibit RIG-I function [16,46]. LMP1 is EBV's primary oncogenic protein. LMP1 disrupts a wide array of cellular mechanisms to promote oncogenic transformation, angiogenesis, cell proliferation and survival, metastasis and invasion, and aids an immunosuppressive tumour microenvironment [47]. Lu et al. suggest that LMP1 interacts with an E3 ligase and degrades RIG-I via the proteasome pathway in an NPC cell line [46]. LMP1 was reported to reduce RIG-I and MDA5 expression, and suppress IFN-B and ISRE promoter activation, IRF3 phosphorylation and ISG15 expression mediated by Sendai virus in a C666-1 cell line [46]. However, the mechanism is not explained and is contrast to a previous report of LMP1 priming IFN production [48].

### 4.3 BRLF1 reduces transcription of RIG-I activators, prevents RIG-I inflammasome activation

BRLF1 is an immediate-early EBV protein which plays a key role with BZLF1 in activating viral transcription and lytic replication. BRLF1 is expressed in the early lytic stage and is reported to inhibit RIG-I-dependent inflammasome activation and pyroptosis [16]. Inflammasomes are constitutively activated in latent herpesvirus infection and inflammasome activation is suppressed during lytic replication [16]. RIG-I inflammasome can facilitate IL-1β expression through activation by EBERs [49]. IL-1β inhibits tumour growth in mice, enhancing survival due to local anti-tumour immunity dependent on tumour-associated neutrophils (TANs) in the tumour microenvironment. Tumour-derived IL-1β enhances infiltrating TANs, inhibiting tumour growth, and improving patient survival [49]. Patients with EBV-associated NPC with higher expression of RIG-I, AIM2 and NLRP3 inflammasomes correlated with better patient survival [49]. BRLF1 is reported to suppress inflammasome activation through interaction with RNA Pol III subunits, suppressing the RNA Pol III-mediated production of 5'ppp RNA and transcription of viral small RNAs such as EBERs (HSV-1 LAT). In human lymphocytes during primary infection and lytic lifecycle, EBV BRLF1 allows immune evasion of T and NK cells by preventing RIG-I-inflammasome activation [16]. EBV BRLF1 disrupts RIG-I signaling to promote viral and tumour immune escape and contributes to a tumour permissive microenvironment, it is an important mechanism EBV uses to prevent RIG-I activation during early lytic cycle.

### 4.4 BHRF1 disrupts RIG-I signaling molecule, MAVS, to prevent apoptosis

While EBV BRLF1 inhibits RIG-I activation by selectively controlling protein production, another EBV protein, BHRF1 (Bam H Rightward open reading frame 1) disrupts mitochondrial antiviral signaling protein (MAVS), inhibiting the RLR signaling pathway and type I IFN activation as a mechanism of innate immune evasion [50].

Herpesviruses have evolved viral homologs of human proteins, around 10% of EBV proteins share homology with human proteins, and that rises to around a third of KSHV proteins [5]. EBV BHRF1 and KSHV KS-Bcl-2 (ORF16) are viral homologs for the human anti-apoptotic regulator BCL-2. Although the human and viral proteins share homology, they can differ in function. It has been reported that G-quadruplexes in the regulatory regions of BHRF1 and KS-Bcl-2 promote transcription, yet the G-quadruplexes in BC-L2 regions inhibit transcription [51].

BHRF1 and KS-Bcl-2 are expressed in early lytic replication, with BHRF1 also expressed in some EBV latency phases [50]. BHRF1 prevents apoptosis in primary infection and BHRF1 also documented to subvert autophagy to improve viral replication [50,52]. BHRF1 is also primarily located in the mitochondrial membrane and shares anti-apoptotic properties through its interactions with pro-apoptotic BAK1 and BH3-only proteins. BHRF1 interacts with the autophagic machinery protein BECN1/Beclin to induce accumulation of autophagosomes beginning mitophagy. BHRF1 inhibits type I IFN expression through mitochondrial degradation and disrupting the function of MAVS [50].

To summarise, EBV has methods for both the promotion and prevention of RIG-I activation. EBERs and circBART2.2 bind and directly activate RIG-I signaling inducing the expression of autocrine growth factors, pro-inflammatory cytokines and the exclusion of tumour killing T and NK cells [10,14,28]. RIG-I activation preventors, BART6-3p and LMP1, can directly bind and degrade RIG-I and BRLF1 prevents transcription of host and viral RIG-I activators [16,17,46,49]. EBV BHRF1 inhibits MAVS through mitophagy process and promotes antiapoptotic pathways [50].

## 5 EBV and KSHV have conserved mechanisms of inhibiting the RIG-I regulator TRIM25

Herpesviruses have conserved functions across species. KSHV ORF64, EBV BPLF1, HCMV-UL48 and HSV-1 UL36 encode for lytically expressed viral tegument proteins with low sequence homology but similar deubiquitinating activity capable of cleaving Lys48-or Lys63-linked polyubiquitin chains [15,53,54]. These homologs can inhibit RIG-I activation by interacting with RLR regulators. TRIM25 is a positive regulator of RLR signaling and activates RIG-I via ubiquitination. KSHV ORF64 and EBV BPLF1 inhibit TRIM25-mediated ubiquitination of RIG-I [15,53]. EBV BPLF1 and KSHV ORF64 are recruited to TRIM25 ligase and 14-3-3 molecular scaffold, resulting in TRIM25 sequestered in the cytoplasm [53,54]. RIG-I function is inhibited by KSHV ORF64 deubiquitylation. ORF64 deubiquitinates RIG-I-2-CARD thus inhibiting IRF3 activation [15].

Curiously, the HSV-1 UL36 interacts with TRIM25 but does not have efficient interaction on the 14-3-3 molecular scaffold to allow sequestration in the cytoplasm [53]. HSV-UL36 and HCMV-UL48 disrupt the ubiquitination of TRAF3 [53]. Although viral homologs with a conserved immune evasion function, the immune target differs with their preferred cell host. Both oncogenic γherpesviruses achieve this function with a common host immune target. By disrupting TRIM25, EBV and KSHV limit the innate response and inhibit type I IFN expression.

## 6 KSHV inhibits interferon regulatory factors (IRFS) activation to escape interferon (IFN) response

Host interferon regulatory factors (IRFs) are transcription factors indispensable for the induction of IFNs, (namely IRF3, IRF7 and IRF9), and known to play a role in other key cellular processes such as cell growth, differentiation and apoptosis [55]. Interestingly, KSHV encodes 4 viral homologs of cellular IRFs, vIRF1-4 [56]. These vIRFs have a certain degree of homology to human IRFs. However, they do not contain key amino acids in their DNA-binding region and are not able to bind to the promoters of type I IFN or ISGs. As discussed previously, lytic stage KSHV activates RIG-I through direct binding of KSHV genomic fragments and allowing the accumulation of host vtRNAs [12,13]. RIG-I activation induces phosphorylation/translocation of the transcription factors, IRF3 and IRF7, to induce type I IFN expression. A novel mechanism for viral immune evasion was reported by Zhu et al., 2002, that a viral protein (ORF45) could inhibit type I IFN induction by blocking the activation and translocation of a transcription factor, IRF7 [57]. ORF45 efficiently inhibits expression of IFN-α and IFN-β during viral infection. Thus, ORF45 allows KSHV to evade KSHV-activated RIG-I immune response by blocking expression of IFNs.

## 7 KSHV manipulates interferon stimulated genes (ISGs) to pro-viral factors

Induction of IFNs and ISGs via RLR pathways are a key part of the anti-viral immune response. KSHV modulates the function of antiviral ISGs to aid lytic replication. KSHV ORF44 and ORF20 proteins interact with ISGs to control genome replication and protein production, respectively [58,59]. Interestingly, KSHV hijacks antiviral ISG proteins, Viperin and 2′-5′-oligoadenylate synthetase-like protein (OASL), to pro-viral factors [58,59].

KSHV ORF44 is a lytically expressed helicase that functions to initiate viral DNA replication [59]. Helicases, RIG-I and ORF44 for example, require post-translational modifications (PTMs), such as methionine oxidation, for biological stability and function [59]. Methionine oxidation is catalysed by the ISG protein Viperin [59]. Viperin is essential for KSHV genome replication, is upregulated by KSHV RTA (ORF50) and is the most abundant ORF44 binding protein. Bai et al.*, reports that* Viperin catalyses methionine oxidation of ORF44 and RIG-I to promote helicase stability, half-life, and protein expression [59]., KSHV hijacks host antiviral response to control host and viral DNA replication initiation. *.*

KSHV ORF20 can interfere with an antiviral ISG product, OASL, to selectively control protein production [58]. OASL prevents viral replication of some viruses, but not all, and is classed as an ISG with targeted antiviral specificity. OASL and KSHV-ORF20 co-localize in nucleoli and are reported by Bussey et al. to associate and purify with cellular translational machinery components [58]. OASL mRNA expression was upregulated downstream of RIG-I in an IRF3-dependent manner during lytic reactivation of latent cells [58]. It is worth noting that the KSHV protein that upregulates Viperin which stabilises the RIG-I helicase, and the KSHV and host derived RNAs that activate RIG-I are both expressed in the lytic stage. Activated RIG-I signaling induces expression of IFNs and ISG proteins, including Viperin. The role of RIG-I activation in the lytic cycle of KSHV deserves further study, to identify potential target cytokines or chemokines that the virus may utilise to further viral or tumour progression. KSHV potentially has further mechanisms to evade RIG-I and MDA5 during latency, and knowledge of methods to disrupt KSHV latency without inducing lytic reactivation may have therapeutic potential.

To sum up, KSHV encodes 3 conserved genomic fragments that bind to RIG-I, KSHV manipulates cellular pathway to allow the accumulation of host dsRNA that binds to RIG-I, and RIG-I activation is lytic life cycle dependent. KSHV has conserved methods of activating RIG-I. KSHV has methods of viral immune evasion to aid lytic replication by controlling DNA replication and protein production [58]. KSHV activates RIG-I and has methods of avoiding the antiviral effect and hijacking ISGs to pro-viral factors. KSHV has methods of avoiding the antiviral effects of the RIG-I pathway through disruption of IRFs, and swaying ISG proteins to pro-viral factors.

## 8 RIG-I in EBV associated malignancies

RIG-I may have a protective role in classical Hodgkin lymphoma (cHL) in young patients yet support an immunosuppressive environment in older patients [60,61]. RIG-I single nucleotide polymorphism (SNP) have been associated with nodular sclerosis in EBV-positive younger age cHL patients [61]. While Satoh et al. investigated RIG-I signaling in EBV-positive and EBV-negative cHL elderly patients found although RIG-I expression increased, no increase in IRF3 activation or IFN-β expression was reported in EBV-positive elderly cHL patients [60]. EBV-positive cHL elderly patients had increased levels of FOXP3^+^ T cells and reduced expression of granzyme B when compared to EBV-negative patients. CCL20 levels, an inhibitory cytokine, were increased in EBV + cHL cells. Satoh et al. suggest that EBV latent infection modifies chemokine expression, the innate immune response leading to an immunosuppressive TME and an adverse outcome in CHL of the elderly [60].

RIG-I plays a critical role mediating the antitumour response after chemotherapy or radiotherapy. The detection of cytosolic RNA and DNA, resulting from double-stranded breaks (DSB) induced by radiotherapy, is dependent upon RIG-I/MAVS and cGAS-STING pathways [62]. These activated cytosolic immunoreceptors lead to a type I IFN-mediated innate and adaptive response which is essential for efficacy of radiotherapy [62,63]. Jing et al. investigated the role of RIG-I in NPC resistant to both radiotherapy and chemotherapy and found that RIG-I expression was significantly reduced in chemoradiotherapy-resistant NPC tissues and cells [64]. Overexpression of RIG-I increased sensitivity to both therapies, accompanied with an increase in IFN/JAK2 and endoplasmic reticulum (ER) stress response markers [64]. As discussed previously, the RIG-I response pathway is manipulated by EBERs to promote tumour growth in NPC [14,28,41]. Viral mechanisms of promoting RIG-I activation can enhance tumour growth and prevention of RIG-I-mediated antiviral responses can enhance therapy resistance.

Numerous studies are underway investigating how to harness immunogenic cell death induced by cancer therapies. For example ataxia-telangiectasia-mutated (ATM) and Rad3-related protein kinase (ATR) is a key regulator of the DNA damage response and ATR inhibitors are of interest to cancer researchers to potentiate the effects of radiotherapy [62,65]. Understanding if response requires functional RIG-I, undisturbed by viral mechanisms, will be important for widespread success of both these agents and other vaccines and immunotherapies [66] in virally associated cancers.

## 9 Future for RIG-I as a potential immunotherapeutic for viral induced cancers

RIG-I is expressed in various cell types, including tumour cells [67]. RIG-I activation can lead to apoptosis or pyroptosis of tumour cells, recruitment of immune cells, and aid presentation of tumour-associated antigens to the adaptive response [68,69]. Iurescia et al. *2020,* reviewed methods of inducing tumour cell death and relevant clinical trials involving RIG-I agonists [70]. Therapies that take advantage of anti-tumour RLR responses include synthetic RIG-I agonists, oncolytic viruses, and epigenetic drugs.

MK-4621/RGT100 is a synthetic RIG-I agonist that has been trialled in two phase I studies as a monotherapy (NCT03065023) and as a combination therapy with the PDL1 checkpoint inhibitor pembrolizumab (NCT03739138) [71]. MK-4621 was found to have tolerable safety in advanced stage solid tumours, RIG-I was activated with an increase in IFN signaling pathway members, however, there was modest antitumour activity. It was concluded there was no meaningful clinical benefit at the doses tested [71]. CV8102/RNAdjuvant® is a 547 nt, noncoding, uncapped ssRNA-based immunomodulator containing polyU-repeats complexed with a polymeric carrier formed by disulfide-crosslinked cationic peptides [72]. Originally trialled as a rabies vaccine adjuvant (NCT02238756), CV8102 has been trialled for use as an intratumoral injection to stimulate a T-helper type 1 cell (Th1) response by activating TLR7/8 and RLRs [73,74]. CV8102 combined with multipeptide antigens (IMA970A) were investigated in a phase I/II cancer vaccine trial (NCT03203005) for hepatocellular carcinoma [74]. The vaccine, named HepaVac-101, showed a favourable safety profile and moderate immunogenicity [74]. CV8102 trials have been expanded to evaluate antitumour response in patients with advanced melanoma, squamous cell carcinoma of the skin, squamous cell carcinoma of the head and neck, or adenoid cystic carcinoma (NCT03291002) [75]. CV8102 will be given as a monotherapy or in combination with standard of care anti-PD-1 therapy with results expected in 2023.

Oncolytic viruses are a promising avenue for cancer treatments and is a rapidly growing field of interest, particularly since 2015 when the FDA approved the first oncolytic virus Talimogene laherparepvec (Imlygic®; or T-VEC) for treatment of melanoma [76]. Oncolytic therapies are thought promising for the effects to enhance sensitivity to immune check-point inhibitors [77]. Hemagglutinating virus of Japan-envelope (HVJ-E/GEN0101) is an oncolytic virus that was trialled for treatment in patients with castration resistant prostate cancer (UMIN000017092) and advanced melanoma (UMIN000012943) [78,79]. HVJ-E is an inactivated form of HVJ, also known as Sendai virus, and is a member of the ssRNA Paramyxovirus family. HVJ-E has a multifaceted method of inducing apoptosis of tumour cells, but as the virus is inactivated via ultraviolet irradiation, the ssRNA fragments are shown to activate the RIG-I pathway and induce type I IFN response [80]. In the phase I trials (UMIN000017092 and UMIN000012943) GEN0101 was administered as an intratumoral injection and was found to be safe, tolerable and exert anti-tumour effects [78,79]. From these promising results, GEN0101 is currently being trialled as a combination therapy with Pembrolizumab for patients with advanced melanoma (NCT03818893) [79]. GEN0101 is also being trialled in patients with chemotherapy-resistant malignant pleural mesothelioma (UMIN000019345) [81].

Epigenetic drugs are found to kill cancer cells by not only preventing methylation of tumour-associated genes, also by inducing an effect in tumour cells named ‘viral mimicry’ [70]. DNA methyltransferase inhibitors (DNMTis) have been reported to kill cancer cells since the seventies, however the intricate the mechanism of action was poorly understood [[82], [83], [84]]. 5-azacytidine (azacytidine, AZA) and it's derivative 5-2′-deoxycytidine (decitabine, DAC) are FDA approved for patients with the pre-leukemic disorder, Myelodysplastic syndrome. Azacytidine is reported to induce anti-tumour effects through the activation of RNA sensors RIG-I/MAVS and the TLR3 pathways in ovarian, and colorectal cancers [85,86]. However, AZA has been reported to induce lytic reactivation of EBV [[87], [88], [89]]. Zebularine is a DMNTi investigated that did not induce EBV lytic reactivation, and therefore may be a more suitable option in the future for EBV-related malignancies [88].

RIG-I agonists have been used successfully in combination with other immunotherapies to kill tumour cells. RIG-I in combination with CTLA-4 checkpoint inhibitors have been used to treat experimental melanoma in mice [68]. RIG-I activation and CTLA-4 blockade can prime and activate cytotoxic T cells to kill tumour cells [68]. Short interfering RNA (siRNA) with 5'ppp-moeity has been used to silence Bcl-2 and activate RIG-I leading to apoptosis of tumour cells in a murine model of lung metastases [69]. The tumour microenvironment is essential to determine the success of immunotherapies. RIG-I-mediated apoptosis of tumour cells releases DAMPs and chemokines in the tumour microenvironment which recruits and activates cytotoxic CD8^+^ T cells and Natural killer (NK) cells [68]. The immunosuppressive tumour microenvironment is changed into an immunostimulatory TME, enhancing tumour killing, inhibiting tumour growth, and potentially enhancing treatment outcome.

Oncogenic y-herpesviruses alter the innate response and may prevent the RIG-I-mediated apoptosis of cancer cells. Potential immunotherapies for virally induced cancers must restore function to RIG-I before seeking to harness the RIG-I-mediated antitumour effects. The widening knowledge of viral mechanisms of immune evasion will bring deeper insight into how the immune system may be channelled to aid future treatment options for patients with virally associated cancers.

## Funding

AN is funded by a Faculty of Science and Engineering Scholarship from the 10.13039/501100001635University of Limerick.

## Author contribution statement

AN and EJR conceived and designed the review. AN reviewed the literature, drafted the manuscript and prepared the figure and table. EJR revised the manuscript. Both authors approved the final version.

## Declaration of competing interest

The authors declare that they have no known competing financial interests or personal relationships that could have appeared to influence the work reported in this paper.
